# Protein Phosphatase 5 Promotes SUMM2-Mediated Immunity by Facilitating Its Protein Accumulation

**DOI:** 10.3390/plants15121875

**Published:** 2026-06-17

**Authors:** Xingchuan Huang, Yanan Liu, Yuelin Zhang

**Affiliations:** 1 Key Laboratory of Regional Characteristic Agricultural Resources, College of Life Sciences, Neijiang Normal University, Neijiang 641100, China; 10001997@njtc.edu.cn; 2 Key Laboratory of Bio-Resource and Eco-Environment of Ministry of Education, College of Life Sciences, Sichuan University, Chengdu 610065, China; yanan.liu@scu.edu.cn; 3 Department of Botany, University of British Columbia, Vancouver, BC V6T 1Z4, Canada

**Keywords:** protein phosphatase 5 (PP5), SUMM2, HSP90, chaperone

## Abstract

Protein Phosphatase 5 (PP5) is an evolutionarily conserved serine/threonine phosphatase with a unique tetratricopeptide domain. It has been implicated in a wide range of cellular processes in mammals, but its function in plants is unknown. Here, we uncovered that *Arabidopsis* PP5 is required for immunity mediated by the nucleotide-binding leucine-rich repeat immune receptor protein SUMM2. Loss-of-function mutations in *PP5* suppress the autoimmune phenotypes caused by the activation of SUMM2 due to the disruption of the MEKK1-MKK1/MKK2-MPK4 kinase cascade. Further biochemical analysis revealed that SUMM2 interacts with Heat Shock Protein 90 (HSP90) and PP5, and SUMM2 level is reduced in *pp5* knockout mutant plants, suggesting that PP5 promotes the accumulation of SUMM2, likely through its association with the HSP90 chaperone complex.

## 1. Introduction

To defend themselves against infections from various microbial pathogens, plants have evolved sophisticated immune systems [1,2]. Pathogen-associated molecular pattern (PAMP)-triggered immunity (PTI) is initiated by recognizing conserved microbial features through cell surface-localized pattern recognition receptors (PRRs). Activation of PTI is believed to be sufficient to stop most pathogen infections. However, adapted pathogens have evolved effector proteins to inhibit PTI and promote colonization. Recognition of pathogen effectors by plant resistance (R) proteins leads to activation of effector-triggered immunity (ETI). Most R proteins identified so far are nucleotide-binding leucine-rich repeat (NLR) proteins with either a Toll/Interleukin-1 receptor (TIR) or a coiled-coil (CC) domain at their N-termini [3]. Recognition of effectors by R proteins can be directly through physical interaction or indirectly through detecting effector-mediated modification of host proteins [4]. PTI and ETI are functionally interdependent, mutually potentiating each other to establish robust plant immunity [5,6,7,8].

In *Arabidopsis*, the MEKK1–MKK1/MKK2–MPK4 mitogen-activated protein (MAP) kinase cascade constitutes a key signaling module in plant innate immunity [9,10]. This cascade is essential not only for basal resistance against pathogens but also for maintaining immune homeostasis, as its integrity is actively monitored by the CC-type NLR (CNL) SUMM2 [11]. Consequently, loss of MEKK1, MKK1/MKK2, or MPK4 leads to constitutive, SUMM2-dependent activation of defense responses, indicating that SUMM2 detects disruption of this cascade. Mechanistically, SUMM2 physically interacts with CALMODULIN-BINDING RECEPTOR-LIKE CYTOPLASMIC KINASE 3 (CRCK3), a direct substrate of MPK4 [12]. In wild-type plants, MPK4-mediated phosphorylation of CRCK3 keeps the protein in a state that prevents SUMM2 activation. When the MEKK1–MKK1/MKK2–MPK4 cascade is compromised, CRCK3 phosphorylation is reduced, and this hypophosphorylated state is sensed by SUMM2 as a “modified-self” signal, thereby triggering downstream immune responses. Thus, CRCK3 functions as a classical guardee, coupling the integrity of this MAP kinase cascade to NLR-mediated immunity.

The establishment of robust NLR-mediated immune responses hinges on proper accumulation of NLR immune receptors, which are tightly controlled by the molecular chaperone network. Heat Shock Protein 90 (HSP90), a family of highly conserved molecular chaperones, promotes the folding of client proteins and is indispensable for plant immunity. In *Arabidopsis*, cytosolic HSP90s, together with the co-chaperones RAR1 (Required for MLA12 resistance 1) and SGT1 (Suppressor of the G2 allele of Skp1), are required for the proper accumulation and/or folding of multiple NLRs, including MLA, RPM1, RPS2, RPS4, N, and I2 [13,14,15,16,17,18]. Intriguingly, certain mutations in HSP90.2 and HSP90.3 lead to increased rather than decreased levels of SNC1, RPS2, and RPS4 [14,19], suggesting that HSP90s also assist SGT1 in the assembly of SCF E3 ubiquitin ligase complexes to promote the degradation of specific NLRs. Thus, the HSP90 chaperone machinery can both stabilize and destabilize NLR proteins, and its functional output is likely dictated by distinct sets of co-chaperones. However, whether SUMM2 is regulated by a specific co-chaperone remains unknown.

In this study, we report the identification and characterization of SUMM5, a positive regulator of SUMM2-mediated immunity. SUMM5 was isolated from a modified suppressor screen of the *mkk1 mkk2* double mutant, and loss-of-function mutations in SUMM5 partially suppressed the autoimmune phenotypes of *mekk1*, *mkk1 mkk2*, and *mpk4* plants. Positional cloning revealed that SUMM5 encodes Protein Phosphatase 5 (PP5). Further mechanistic analyses demonstrated that SUMM2 physically associates with HSP90 and PP5, and that PP5 likely functions as an HSP90 co-chaperone to regulate the proper accumulation of SUMM2. Importantly, bacterial growth assays showed that the *pp5* mutant supported wild-type levels of *Pseudomonas syringae pv. tomato* DC3000 carrying AvrRpt2, AvrRPS4, AvrPphB, or the T3SS-deficient *hrcC* mutant strain, indicating that PP5 does not detectably affect RPS2-, RPS4-, RPS5-, or PTI-mediated resistance. Collectively, these findings suggest that PP5/SUMM5 likely serves as an HSP90 co-chaperone that specifically couples the chaperone network to SUMM2-mediated surveillance of the MEKK1–MKK1/MKK2–MPK4 kinase cascade, without broadly influencing other NLR signaling pathways.

## 2. Results

### 2.1. Identification and Characterization of summ5 mkk1 mkk2 ndr1

**Figure 1:**
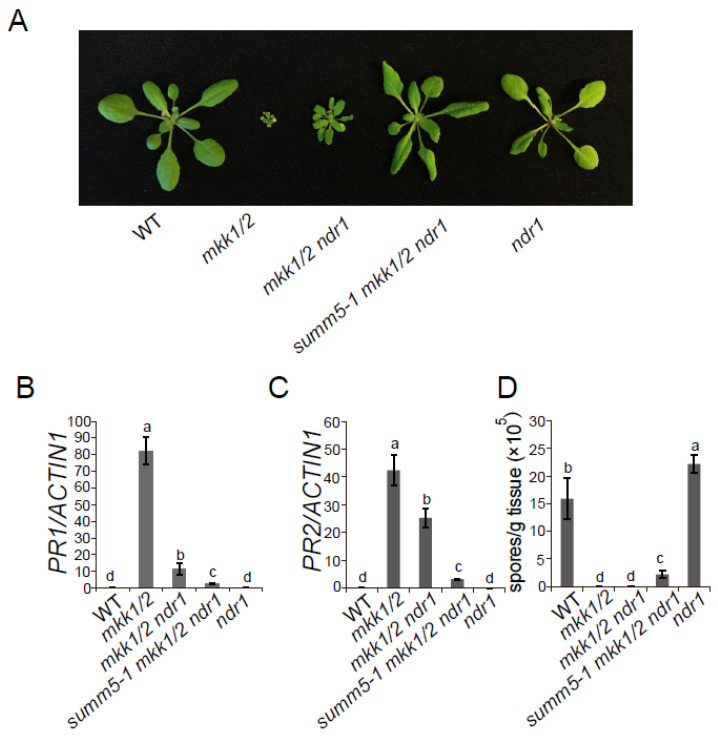
Characterization of the *summ5-1 mkk1-1 mkk2-1 ndr1-1* mutant. (**A**) Morphology of three-week-old soil-grown wild-type (WT) Col-0, *mkk1-1 mkk2-1* (*mkk1/2*), *mkk1-1 mkk2-1 ndr1-1* (*mkk1/2 ndr1*), *ndr1-1* (*ndr1*) and *summ5-1 mkk1-1 mkk2-1 ndr1-1* (*summ5 mkk1/2 ndr1*) plants. (**B**,**C**) Expression levels of *PR1* (**B**) and *PR2* (**C**) in WT, *mkk1/2*, *mkk1/2 ndr1*, *ndr1* and *summ5 mkk1/2 ndr1* seedlings. Two-week-old seedlings grown on 1/2 MS plates were used. Values were normalized to the expression levels of *ACTIN1*. (**D**) Growth of *H.a.* Noco2 on WT, *mkk1/2*, *mkk1/2 ndr1*, *ndr1* and *summ5 mkk1/2 ndr1* seedlings. Error bars represent the standard deviations of three measurements. Statistical differences among the samples are labeled with different letters (*p* < 0.01, One-way ANOVA and Tukey’s test; *n* = 3).

In our previous *mkk1 mkk2* suppressor screen, the *mkk1 mkk2* mutant exhibited strong sterility, which severely limited the size of the M_2_ population. Consequently, only SUMM1/MEKK2, SUMM2, SUMM3/CRCK3, and SUMM4/MKK6 were identified from this screen [11,12,20,21]. To identify additional regulators of SUMM2-mediated defense, we extended this screen using a *mkk1-1 mkk2-1 ndr1-1* (*mkk1/2 ndr1*) background, which overcomes the sterility of *mkk1 mkk2* and permits larger-scale screening. In addition to SUMM6/MDS1, which we previously reported from this screen [22], another mutant we characterized is *summ5-1*. As shown in Figure 1A, the *summ5 mkk1 mkk2 ndr1* quadruple mutant is markedly larger than the *mkk1 mkk2 ndr1* triple mutant, although slightly smaller than wild-type. Quantitative RT-PCR (qRT-PCR) analysis revealed that the constitutive expression of *PR1* and *PR2* in *mkk1 mkk2 ndr1* is strongly reduced by *summ5-1* (Figure 1B,C). Moreover, the enhanced resistance of *mkk1 mkk2 ndr1* to the virulent oomycete pathogen *Hyaloperonospora arabidopsidis* (*H. a.*) Noco2 was attenuated by the *summ5-1* mutation (Figure 1D). Together, these data demonstrate that *summ5-1* largely suppresses the autoimmune phenotypes of *mkk1 mkk2 ndr1*.

### 2.2. SUMM5 Encodes PROTEIN PHOSPHATASE 5 (PP5)

**Figure 2:**
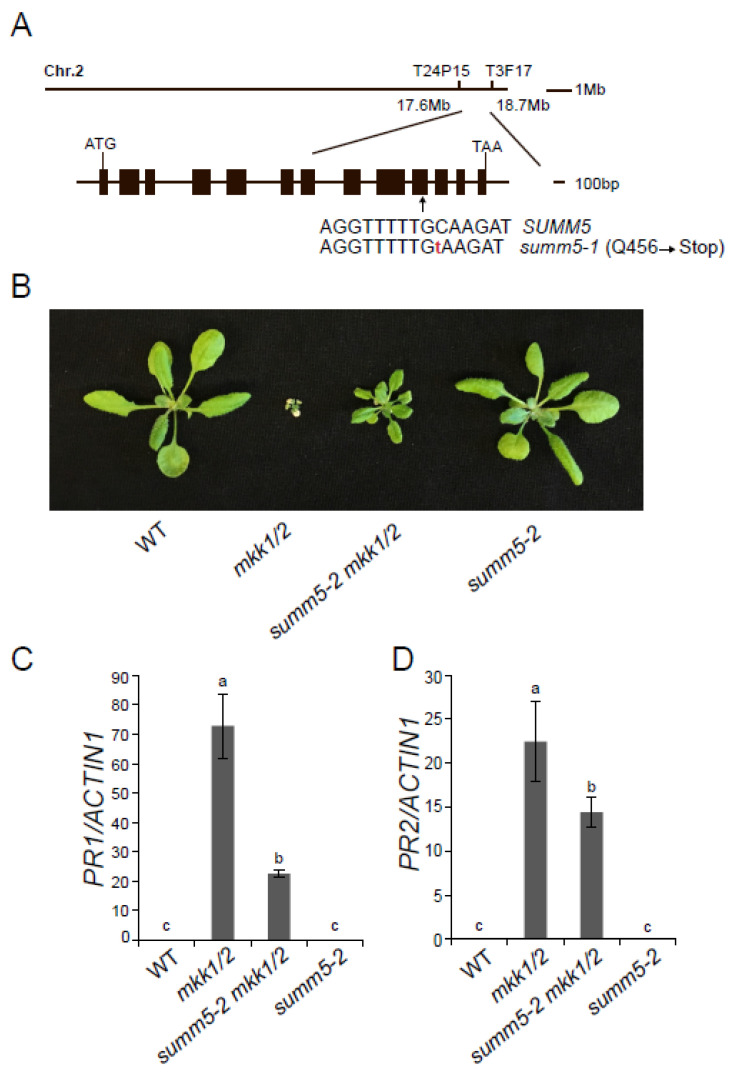
Positional cloning of *SUMM5*. (**A**) Map position and location of the *summ5-1* mutations. (**B**) Morphology of wild-type (WT), *mkk1-1 mkk2-1* (*mkk1/2*), *summ5-2 mkk1-1 mkk2-1* (*summ5 mkk1/2*) and *summ5-2* plants. Plants were grown on soil and photographed three weeks after planting. (**C**,**D**) Expression levels of *PR1* (**C**) and *PR2* (**D**) in WT, *mkk1/2*, *summ5-2 mkk1/2* and *summ5-2* seedlings as compared to *ACTIN1*. Error bars represent the standard deviations of three measurements. Statistical differences among the samples are labeled with different letters (*p* < 0.01, One-way ANOVA and Tukey’s test; *n* = 3).

To map the *summ5-1* mutation, *summ5-1 mkk1 mkk2 ndr1* (in the Col-0 background) was crossed with Landsberg *erecta* (L*er*) to generate a segregating F2 population. A total of 24 plants that are homozygous for *mkk1 mkk2 ndr1* in the F2 progeny were identified and used for crude mapping. *summ5-1* was initially mapped to chromosome 2 between markers T2N18 (15.9 Mb) and F14M4 (19.28 Mb). Further fine mapping narrowed down the mutation to a region between marker T24P15 (17.6 Mb) and T3F17 (18.7 Mb) (Figure 2A). Whole-genome resequencing identified two nonsense mutations in this region, introducing a premature stop codon in *At2g42750* and *At2g42810*, respectively.

To determine which mutation causes the *summ5-1* phenotype, we obtained T-DNA insertional mutants of these two candidate genes from the Arabidopsis Biological Resource Center (ABRC, Columbus, OH, USA). The two T-DNA mutants SALK_051866C (*AT2G42750*) and WiscDsLox368D06/CS864321 (*AT2G42810*) were confirmed to carry insertions in the exons, and therefore were crossed to *mkk1 mkk2*. Only the T-DNA mutant CS864321 partially suppresses the autoimmune phenotype of *mkk1 mkk2* (Figure 2B–D), indicating that *SUMM5* is *AT2G42810.* Thus, we renamed CS864321 *summ5*-2.

To further confirm that the mutation found in *AT2G42810* is responsible for the suppression of *mkk1 mkk2 ndr1* mutant phenotypes, we transformed a construct expressing *AT2G42810* under the control of its native promoter with a C-terminal Green Fluorescent Protein (GFP) tag into *summ5-1 mkk1 mkk2 ndr1*. As shown in Figure S1, the transgene restored the dwarf morphology and elevated *PR1* gene expression of *mkk1 mkk2 ndr1*, further confirming that *SUMM5* is *AT2G42810. AT2G42810* encodes PROTEIN PHOSPHATASE 5 (PP5), which is a unique member of the phosphoprotein phosphatase (PPP) family of serine/threonine phosphatases [23]. It contains a C-terminal phosphatase catalytic domain and an N-terminal tetratricopeptide (TPR) domain.

### 2.3. Defense Responses in mekk1 and mpk4 Are Partially Suppressed by summ5

**Figure 3:**
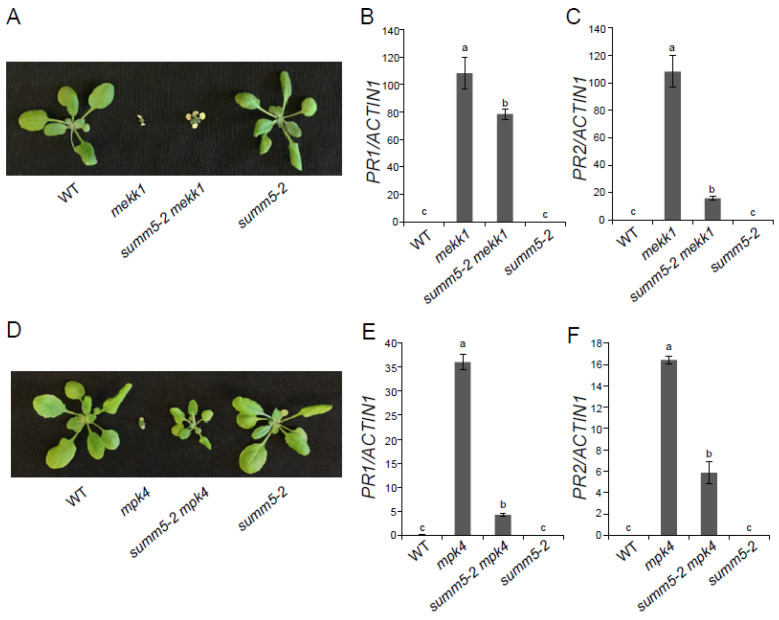
Characterization of the *summ5-1 mekk1-1* and *summ5-1 mpk4-3* double mutants. (**A**) Morphology of three-week-old wild-type (WT), *mekk1-1*, *summ5-1 mekk1-1* and *summ5-1* plants grown on soil. (**B**,**C**) Expression levels of *PR1* (**B**) and *PR2* (**C**) in the indicated genotypes as determined by quantitative RT–PCR. Two-week-old seedlings grown on 1/2 MS plates were used. Values were normalized to the expression levels of *ACTIN1*. (**D**) Morphology of three-week-old WT, *mpk4-3*, *summ5-1 mpk4-3*, and *summ5-1* plants grown on soil. (**E**,**F**) Expression levels of *PR1* (**E**) and *PR2* (**F**) in the indicated genotypes. Two-week-old seedlings grown on 1/2 MS plates were used. Values were normalized to the expression levels of *ACTIN1*. Error bars represent the standard deviations of three measurements. Statistical differences among the samples are labeled with different letters (*p* < 0.01, One-way ANOVA and Tukey’s test; *n* = 3).

As MEKK1, MKK1/MKK2 and MPK4 function in a kinase cascade, we tested whether the autoimmune phenotypes of *mekk1* and *mpk4* can also be suppressed by *summ5*. The *mekk1-1 summ5-2* and *mpk4-3 summ5-2* double mutants were obtained by crossing *summ5-2* with *mekk1-1* and *mpk4-3*, respectively. As shown in Figure 3A, *summ5-2 mekk1-1* is still much smaller than wild type but is considerably bigger than *mekk1-1*. qRT-PCR analysis showed that the elevated expression levels of *PR1* and *PR2* in *mekk1-1* were also partially suppressed by *summ5-2* (Figure 3B,C). Similarly, *summ5-2* partially suppressed the dwarf morphology and constitutive *PR* gene expression in *mpk4-3* (Figure 3D–F). Taken together, these genetic data indicate that SUMM5 functions downstream of the MEKK1-MKK1/MKK2-MPK4 kinase cascade.

### 2.4. PP5 Affects the Accumulation of SUMM2

**Figure 4:**
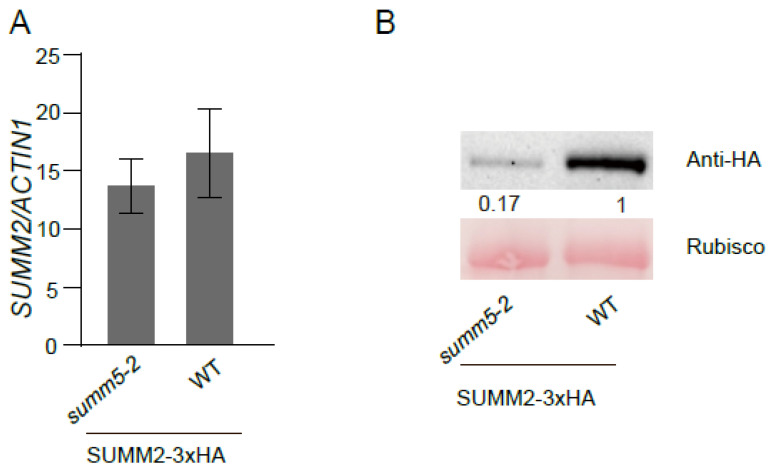
SUMM2 protein level is reduced in *summ5-2*. (**A**) Expression levels of *SUMM2-HA* in wild-type (WT) and *summ5-2* as determined by quantitative RT–PCR. Ten-day-old seedlings grown on 1/2 MS plates were used. Values were normalized to the expression levels of *ACTIN1*. (**B**) Western blot analysis of SUMM2-HA protein levels in WT and the *summ5-2* mutant background. Total protein was extracted from ten-day-old seedlings grown on half-strength MS plates. Rubisco was used as the protein loading control.

In mammals, PP5 functions as a co-chaperone of HSP90 [24]. To determine whether *SUMM5* is required for the accumulation of SUMM2, a homozygous transgenic line expressing *SUMM2-HA* under the control of the *35S* promoter was crossed with *summ5-2*, and *SUMM2-HA*/*WT* and *SUMM2-HA*/*summ5-2* plants were segregated from the F_2_ population. Quantitative RT-PCR analysis confirmed that *SUMM2-HA* transcript levels were comparable between the two genotypes (Figure 4A). In contrast, Western blot analysis showed that SUMM2-HA protein levels were considerably lower in the *summ5-2* background than in the WT background (Figure 4B and Figure S2). These results indicate that PP5 is required for the proper accumulation of SUMM2 at the protein level.

### 2.5. SUMM2 Interacts with PP5 and HSP90 in Planta

**Figure 5:**
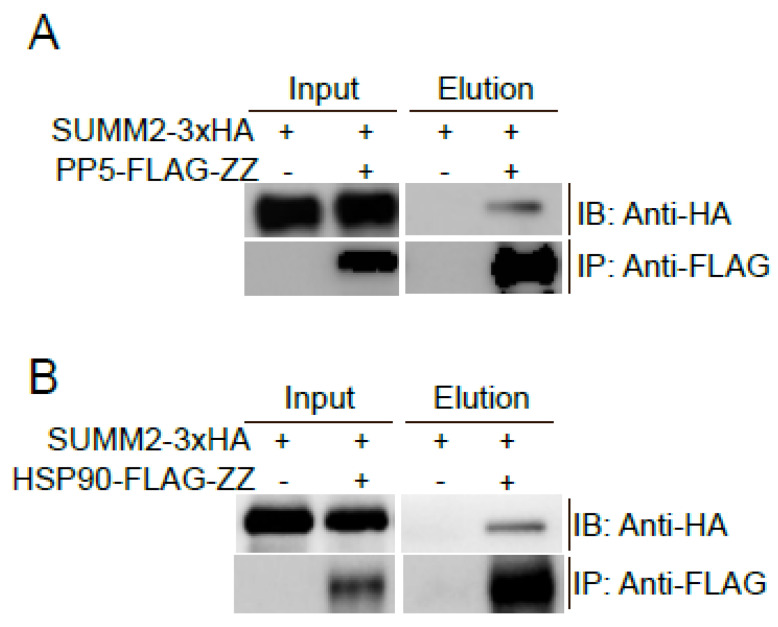
SUMM2 associates with PP5 and HSP90 *in planta.* (**A**) Co-IP analysis to test the interaction between PP5 and SUMM2. PP5-FLAG-ZZ and SUMM2-HA proteins were transiently expressed in *N. bethamiana*. The PP5-FLAG-ZZ protein was immunoprecipitated with anti-FLAG conjugated beads and detected by Western blot using anti-FLAG antibody. The co-immunoprecipitated SUMM2-HA protein was detected by Western blot using anti-HA antibody. Similar results were obtained in three independent experiments. (**B**) Co-IP analysis of interaction between HSP90 and SUMM2. HSP90-FLAG-ZZ and SUMM2-HA proteins were transiently expressed in *N. bethamiana*. HSP90-FLAG-ZZ protein was immunoprecipitated with anti-FLAG conjugated beads and detected by Western blot using anti-FLAG antibody. SUMM2-HA protein was detected by Western blot using anti-HA antibody. Similar results were obtained in three independent experiments.

As PP5 affects SUMM2 accumulation, we further tested whether SUMM2 interacts with PP5. We transiently expressed HA-tagged SUMM2 together with FLAG-ZZ-tagged PP5 in *Nicotiana (N.) benthamiana* by *Agrobacterium*-mediated transformation and carried out immunoprecipitation using anti-FLAG beads. As shown in Figure 5A, SUMM2-HA co-immunoprecipitated with PP5-FLAG-ZZ, suggesting that SUMM2 and PP5 associate with each other *in planta*. We further tested interaction between HA-tagged SUMM2 and FLAG-tagged HSP90 in *N. benthamiana*. As shown in Figure 5B, SUMM2-HA co-immunoprecipitated with HSP90-FLAG-ZZ, suggesting that SUMM2 also associates with HSP90 in vivo.

### 2.6. Mutation of the Conserved Catalytic Histidine Does Not Impair PP5 Function in SUMM2-Mediated Defense

**Figure 6:**
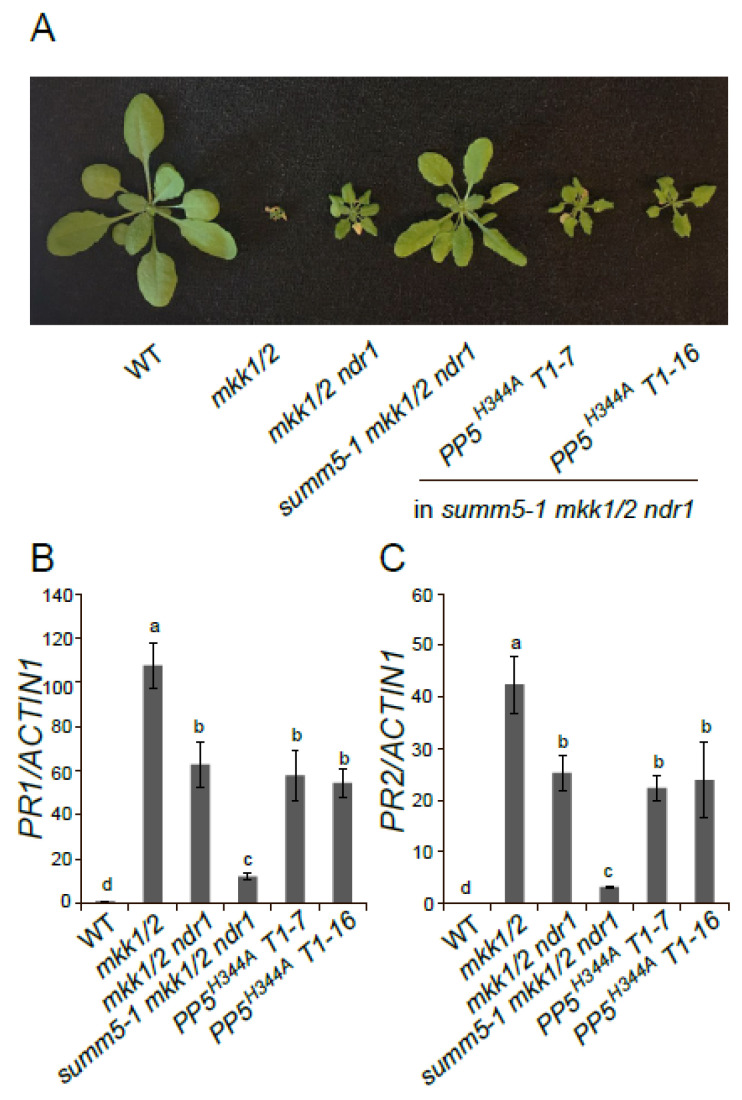
A conserved His (H344) residue in the phosphatase domain is not required for the function of PP5 in the activation of defense responses in *mkk1 mkk2 ndr1*. (**A**) Morphology of three-week-old wild-type (WT), *mkk1-1 mkk2-1* (*mkk1/2*), *mkk1-1 mkk2-1 ndr1-1 (mkk1 mkk2 ndr1)*, *summ5-2 mkk1 mkk2 ndr1* and *PP5^H344A^* transgenic lines grown on soil. PP5^H344A^ T1-7 and PP5^H344A^ T1-16 are independent transgenic lines expressing the PP5^H344A^ mutant protein in the *summ5-2 mkk1 mkk2 ndr1* background. (**B**,**C**) Expression levels of *PR1* (**B**) and *PR2* (**C**) in the indicated genotypes. Total RNA was extracted from two-week-old seedlings grown on 1/2 MS medium. Values were normalized to the expression levels of *ACTIN1*. Error bars represent the standard deviations of three measurements. Statistical differences among the samples are labeled with different letters (*p* < 0.01, One-way ANOVA and Tukey’s test; *n* = 3).

As dephosphorylation of CRCK3 leads to activation of SUMM2-mediated immune responses and PP5 is required for this activation, we initially speculated that PP5 might directly dephosphorylate CRCK3. However, we were unable to detect a direct interaction between CRCK3 and PP5 by co-immunoprecipitation (Co-IP) in *N. benthamiana*. To further test whether the phosphatase activity of PP5 is involved in the regulation of SUMM2-mediated defense signaling, we mutated the conserved histidine residue (H344) in the catalytic domain of PP5 to alanine. This residue corresponds to H304 in human PP5, which has been shown to be essential for phosphatase activity (Figure S3) [25]. We then generated transgenic lines expressing PP5^H344A^ in the *summ5 mkk1 mkk2 ndr1* background. To our surprise, the PP5^H344A^ variant restored the dwarf morphology of *mkk1 mkk2 ndr1* (Figure 6A). Analysis of PR gene expression showed that the expression levels of PR1 and PR2 in the PP5^H344A^ transgenic lines are comparable to those in mkk1 mkk2 ndr1 (Figure 6B,C), indicating that PP5^H344A^ can complement the mutant phenotype of summ5. Given that H344 is predicted to be critical for catalysis based on strong evolutionary conservation and prior biochemical studies in other organisms, these results suggest that the phosphatase activity is largely dispensable for the role of PP5 in regulating SUMM2-mediated defense. We cannot, however, rule out the possibility that a very low level of residual phosphatase activity persists in the H344A variant or that other structural features of PP5 are sufficient to support this specific immune function.

### 2.7. RPS4, RPS2- and RPS5-Mediated Defense Responses Are Not Affected in summ5 Mutants

To determine whether PP5 is required for resistance conferred by other NLR proteins, we infiltrated WT and *summ5* mutant plants with *Pseudomonas syringae* pv. *tomato* (*P.s.t.*) DC3000 strains carrying *AvrRps4*, *AvrRpt2* or *AvrPphB*, which are recognized by the TIR-type NLR RPS4 and CNLs RPS2 and RPS5, respectively [26,27,28]. As shown in Figure S4, growth of these bacterial strains was comparable in WT and *summ5* plants, suggesting that PP5 is not essential for resistance mediated by RPS4, RPS2 or RPS5.

### 2.8. PP5 Is Not Required for PAMP-Triggered Immunity

To determine whether PP5 is involved in PTI, we infiltrated WT and *summ5* mutant plants with *P.s.t.* DC3000 *hrcC*, a bacterial strain deficient in the type III secretion system often used to test PTI response [29,30]. As shown in Figure S5, the growth of *P.s.t.* DC3000 *hrcC* in *summ5-1* and *summ5-2* leaves was comparable to that in WT plants, suggesting that PP5 is not required for PTI.

## 3. Discussion

We previously established a model in which activation of the CNL SUMM2 depends on the integrity of the MEKK1–MKK1/MKK2–MPK4 kinase cascade, with the phosphorylation status of CRCK3 serving as the key signal [12]. To further understand how SUMM2-mediated immunity is regulated, we performed a suppressor screen in the *mkk1 mkk2 ndr1* background and identified *SUMM5*, which encodes the conserved serine/threonine phosphatase PP5 (Figure 1 and Figure 2). Loss-of-function mutations in *SUMM5/PP5* partially suppress the autoimmune phenotypes of *mekk1*, *mkk1 mkk2*, and *mpk4* mutants (Figure 3), suggesting that SUMM5/PP5 functions as a positive regulator of this immune signaling branch. Loss of *PP5* in *Arabidopsis* reduces SUMM2 protein levels, indicating that PP5 positively regulates SUMM2-mediated immunity by promoting the accumulation of SUMM2.

In animals, PP5 participates in diverse cellular processes, whereas plant PP5 has been linked to light signaling, thermotolerance, and interaction with the HSP90–SGT1–RAR1 chaperone complex. Previous work showed that PP5 can associate with tomato NLR I-2, but its functional relevance remained unclear [18]. Here, we uncover a specific requirement for PP5 in the accumulation and activation of SUMM2. We showed that PP5 interacted with SUMM2 and was required for the accumulation of SUMM2 (Figure 4 and Figure 5). Because the predicted catalytic residue H344 is dispensable for PP5 function (Figure 6 and Figures S3), we propose that PP5 may act as an HSP90 co-chaperone to facilitate SUMM2 accumulation, although we cannot rule out other mechanisms. Unlike other protein phosphatases, PP5 contains a unique N-terminal TPR domain, which is involved in the interaction with other proteins. The TPR region was also shown to interact with its C-terminal phosphatase domain to inhibit its phosphatase activity [31]. Interestingly, mutation of the conserved His residue (H344) in the catalytic domain of PP5 does not impair its ability to complement the *summ5* phenotype (Figure 6), suggesting that the phosphatase activity of PP5 is either not required for its function in promoting the accumulation of SUMM2, or a very low level of phosphatase activity is sufficient in this process. However, we cannot rule out the possibility that transgenic expression of PP5^H344A^ may partially compensate for any functional deficiency through increased protein abundance. During the preparation of this manuscript, an independent study also reported that PP5 directly interacts with SUMM2 and that loss of PP5 function leads to reduced SUMM2 protein accumulation [32]. These observations are fully consistent with our findings and further support the conclusion that PP5 is required for proper SUMM2 protein accumulation. In addition to these shared findings, the published study proposed that PP5 directly dephosphorylates CRCK3 and that its phosphatase activity is required for this regulation. While we were unable to detect a physical interaction between PP5 and CRCK3 in our hands using co-immunoprecipitation assays. It is possible that PP5 may engage distinct protein complexes under different experimental conditions or genetic backgrounds, and that its phosphatase activity becomes essential only in certain scenarios. In our model, the primary requirement for PP5 in SUMM2-mediated immunity is to act as a scaffold that facilitates the accumulation of SUMM2.

Loss of PP5 only partially suppresses the autoimmune phenotypes of *mekk1*, *mkk1 mkk2* and *mpk4*, indicating that it functions as a fine-tuning regulator rather than an absolute determinant of SUMM2 accumulation. Furthermore, *pp5* mutations do not detectably affect resistance mediated by RPS2, RPS4, or RPS5, implying that PP5 is either not required for the biogenesis of these NLRs or its contribution falls below the detection limit of our assays. The contribution of PP5 to immunity mediated by different R proteins may vary depending on the nature of specific NLRs. This also raises the interesting possibility that distinct co-chaperones exhibit NLR-client specificity, thereby adding a layer of regulatory precision to immune receptor homeostasis.

## 4. Materials and Methods

### 4.1. Plant Materials and Growth Conditions

Plants were grown under 16 h light at 23 °C and 8 h dark at 19 °C unless specified. *mekk1-1*, *mkk1-1 mkk2-1*, *mpk4-3* and *ndr1-1* mutants were described previously [9,33]. *mkk1-1 mkk2-1 ndr1-1* was obtained by crossing *mkk1-1 mkk2-1* with *ndr1-1*. The *summ5-1* single mutant was obtained by crossing *summ5-1 mkk1-1 mkk2-1 ndr1-1* with WT plants. *summ5-2* (WiscDsLox368D06) was obtained from ABRC. The *summ5-2 mkk1-1 mkk2-1* triple mutant and the *summ5-2 mekk1-1*, *summ5-2 mpk4-3* double mutants were generated by crossing *summ5-2* with *mkk1-1 mkk2-1*, *mekk1-1* and *mpk4-3*, respectively.

### 4.2. Mutant Characterization and Genetic Mapping

For gene expression analysis, total RNA was extracted from 12-day-old seedlings grown on 1/2 MS medium using EZ-10 Spin Column Plant RNA Mini-Preps Kit (Bio Basic, Inc., Markham ON, Canada). Reverse transcription was conducted with M-MuLV reverse transcriptase (abm, Richmond, BC, Canada). qPCR was carried out using the Takara SYBR Premix Ex TaqII kit(Takara, Kyoto, Japan). The primers used to amplify *PR1*, *PR2*, and *ACTIN1* have been described previously [34]. The primers used to amplify *SUMM2-HA* are SUMM2-RT-F and HA-R (Table S1).

To map the *summ5-1* mutation, the *summ5-1 mkk1-1 mkk2-1 ndr1-1* mutant in the Col-0 background was crossed with L*er* plants to obtain a segregating F2 population. F2 plants with *mkk1 mkk2*-like morphology were used for crude and fine mapping. To identify the *summ5-1* mutation, genomic DNA of *summ5-1 mkk1-1 mkk2-1 ndr1-1* was extracted and sequenced using an Illumina Genome Analyzer. Candidate mutations in this region between markers T24P15 and T3F17 were identified by comparing the whole genome sequencing data with the Arabidopsis Col-0 reference genome sequence.

### 4.3. Plasmid Construction

For transgene complementation, a genomic DNA fragment containing *PP5* was amplified from WT genomic DNA using primers 1305-PP5-Kpn1-F and PP5-Xba1-R (Table S1). The PCR fragment was digested with KpnI/XbaI (New England Biolabs, Whitby, ON, Canada) and inserted into a modified p CAMBIA1305 vector with an in-frame GFP tag at the C-terminus. For constructing the *PP5^H344A^* mutant plasmid, two overlapping *PP5* genomic DNA fragments were amplified using primers 1305-PP5-Kpn1-F and PP5-H344A-R or primers PP5-H344A-R and PP5-Xba1-R, and the *PP5^H344A^* mutant gene was subsequently obtained by overlapping PCR using primers 1305-PP5-Kpn1-F and PP5-Xba1-R and the two above DNA fragments as templates (Table S1). After digestion with KpnI/XbaI, the *PP5^H344A^* DNA fragment was cloned into pCAMBIA1305-FLAGZZ.

For Co-IP analysis in *N. benthamiana*, the genomic DNA of *PP5* was amplified using the primers PP5-Kpn1-F and PP5-Xba1-R and cloned into pCAMBIA1300-35S-FLAGZZ. The genomic DNA of HSP90 was amplified using the primers HSP90.3-KpnI-F and HSP90.3-PstI-R and cloned into pCAMBIA1300-35S-FLAGZZ. The *SUMM2* coding sequence was cloned into the pCAMBIA1300-35S vector with a C-terminal triple HA epitope tag (3×HA; referred to as SUMM2-HA throughout the text and figures) under the control of the CaMV 35S promoter. The 1300-35S-SUMM2-HA plasmids were described previously [12].

### 4.4. Co-IP Analysis

For co-IP experiments in *N. benthamiana*, fresh colonies of *Agrobacterium tumefaciens* GV3101 strains containing pCAMBIA1300-35S-SUMM2-HA, pCAMBIA1300-35S-PP5-FLAGZZ, pCAMBIA1300-35S-HSP90-FLAGZZ or an empty vector were inoculated to Luria broth (LB) media supplemented with appropriate antibiotics. The bacteria were grown at 28 °C for 16 h before collection and resuspension in buffer containing 10 mM MES (pH 5.6), 10 mM MgCl_2_ and 150 μMacetosyringone. *Agrobacteria* suspensions carrying different constructs were mixed in a 1:1 ratio with a final concentration of OD_600_ = 0.6 and kept on the bench for three hours at room temperature. The bacterial mixtures were then infiltrated into leaves of four-week-old *N. benthamiana* plants. For each sample, about 1 g of tissue was harvested from the infiltrated area two days after infiltration.

For co-IP analysis, the tissue was ground in liquid nitrogen and resuspended in 2 volumes of grinding buffer (10% glycerol, 25 mM Tris–HCl pH 7.5, 1 mM EDTA, 150 mM NaCl, 10 mM DTT, 2% PVPP, 1× protease inhibitor cocktail from Roche (Basel, Switzerland)). After the samples were fully lysed at 4 °C, they were centrifuged twice at 15,000× *g* for 10 min. The supernatants were collected and transferred to a new tube containing anti-FLAG-conjugated beads. After incubation for 2 h, the beads were collected by centrifugation and washed four times with the extraction buffer plus 1.5% NP-40. Proteins bound to the beads were subsequently eluted with 100 μL of 0.1 mg/mL 3xFLAG peptide (Sigma-Aldrich, St. Louis, MO, USA) and were subjected to SDS–PAGE and Western blot analysis. Unless otherwise indicated, all other reagents were purchased from Sangon Biotech (Shanghai, China).

### 4.5. Pathogen Infection

For *H.a.* Noco2 infection, two-week-old soil-grown plants were sprayed with spores of *H.a.* Noco2 (5 × 10^4^ spores mL^−1^). Afterwards, the plants were covered with a transparent plastic lid and kept in a growth chamber (18 °C, 12 h light/12 h dark cycles, and ~80% humidity) for seven days. The growth of *H.a.* Noco2 was then quantified as described previously [35].

For infection assays of *P.s.t.* DC3000 *hrc*C and *P.s.t.* DC3000 with different effectors, plants were grown at 22 °C under 12 h light/12 h dark cycles. Two leaves of four-week-old plants were infiltrated with bacterial suspensions in 10 mM MgCl_2_. Leaf discs were collected at 3 days post-inoculation and ground in 10 mM MgCl_2_. The samples were diluted and plated on LB medium. Bacterial colonies were counted 2 days later to determine the bacterial titer.

## Data Availability

All data are presented in the manuscript and Supplementary Materials.

## Supplementary Materials

The following supporting information can be downloaded at: https://www.mdpi.com/article/10.3390/plants15121875/s1, Supplementary Figure S1. Complementation of *summ5-1* by *PP5-GFP*. Supplementary Figure S2. SUMM2 protein abundance in WT and *summ5* mutant. Supplementary Figure S3. Multiple sequence alignment of PP5 orthologs from diverse eukaryotes. Supplementary Figure S4. RPS4-, RPS2- and RPS5-mediated immunity is not affected in *summ5* mutants. Supplementary Figure S5. Growth of *P.s.t.* DC3000 *hrcC* in wild-type (WT) and *summ5* mutants. Supplementary Table S1. Primers used in this study.
