# Supplementary material for: Protein Phosphatase 5 Promotes SUMM2-Mediated Immunity by Facilitating Its Protein Accumulation
Source: Plants (Basel). 2026 Jun 17;15(12):1875. doi: 10.3390/plants15121875 (PMC13307163; doi:10.3390/plants15121875)
Supplement: Supplementary file 1 [file plants-15-01875-s001.zip › plants-4334976-supplementary.pdf]

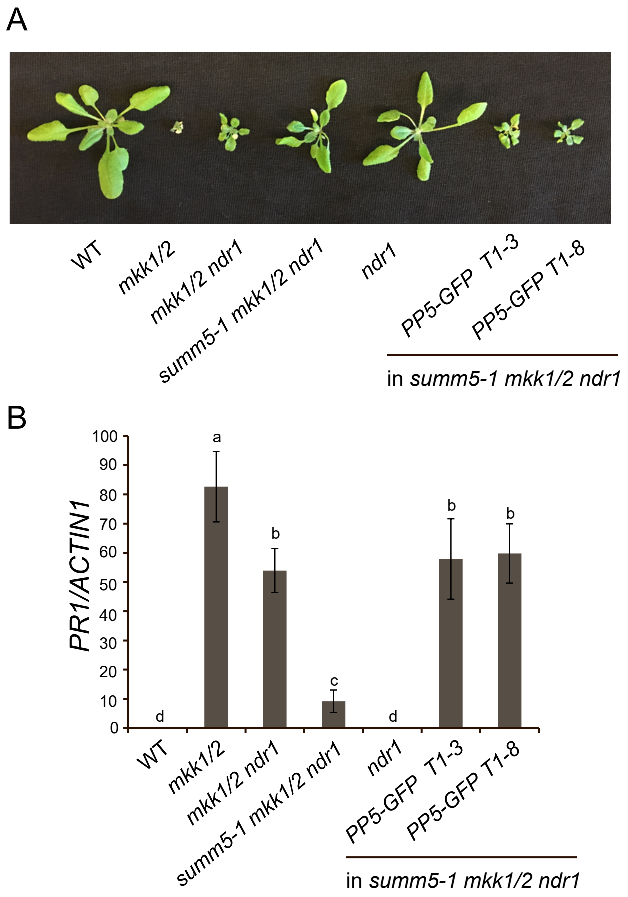

Figure S1. Complementation of *summ5-1* by *PP5-GFP*.
(A) Morphology of wild-type (WT), *mkk1-1 mkk2-1* (*mkk1/2*), *mkk1-1 mkk2-1 ndr1-1* (*mkk1/2 ndr1*), *summ5-1 mkk1-1 mkk2-1 ndr1-1* (*summ5 mkk1/2 ndr1*) and two *PP5-GFP* transgenic lines in *summ5-1 mkk1-1 mkk2-1 ndr1-1* background. The photo was taken with three-week-old soil-grown plants. (B, C) Expression levels of *PR1* (B) and *PR2* (C) in the indicated genotypes as determined by quantitative RT–PCR. Two-week-old seedlings grown on 1⁄2 MS plates were used. Values were normalized to the expression levels of *ACTIN1*.Statistical differences among the samples are labeled with different letters ($P < 0.01$, two-tailed Student's t-test; n=3)

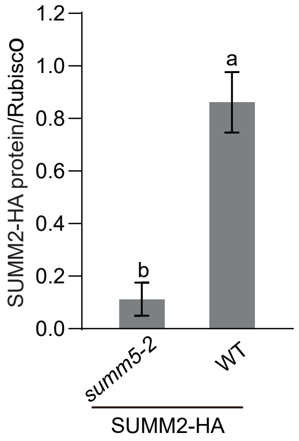

Figure S2. SUMM2 protein abundance in WT and *summ5* mutant.

Densitometric quantification of SUMM2 protein abundance normalized to Rubisco. Relative SUMM2 protein levels are presented as mean ± SD from three independent biological replicates. Statistical differences are labeled with different letters (P < 0.01, two-tailed Student's t-test; n=3)

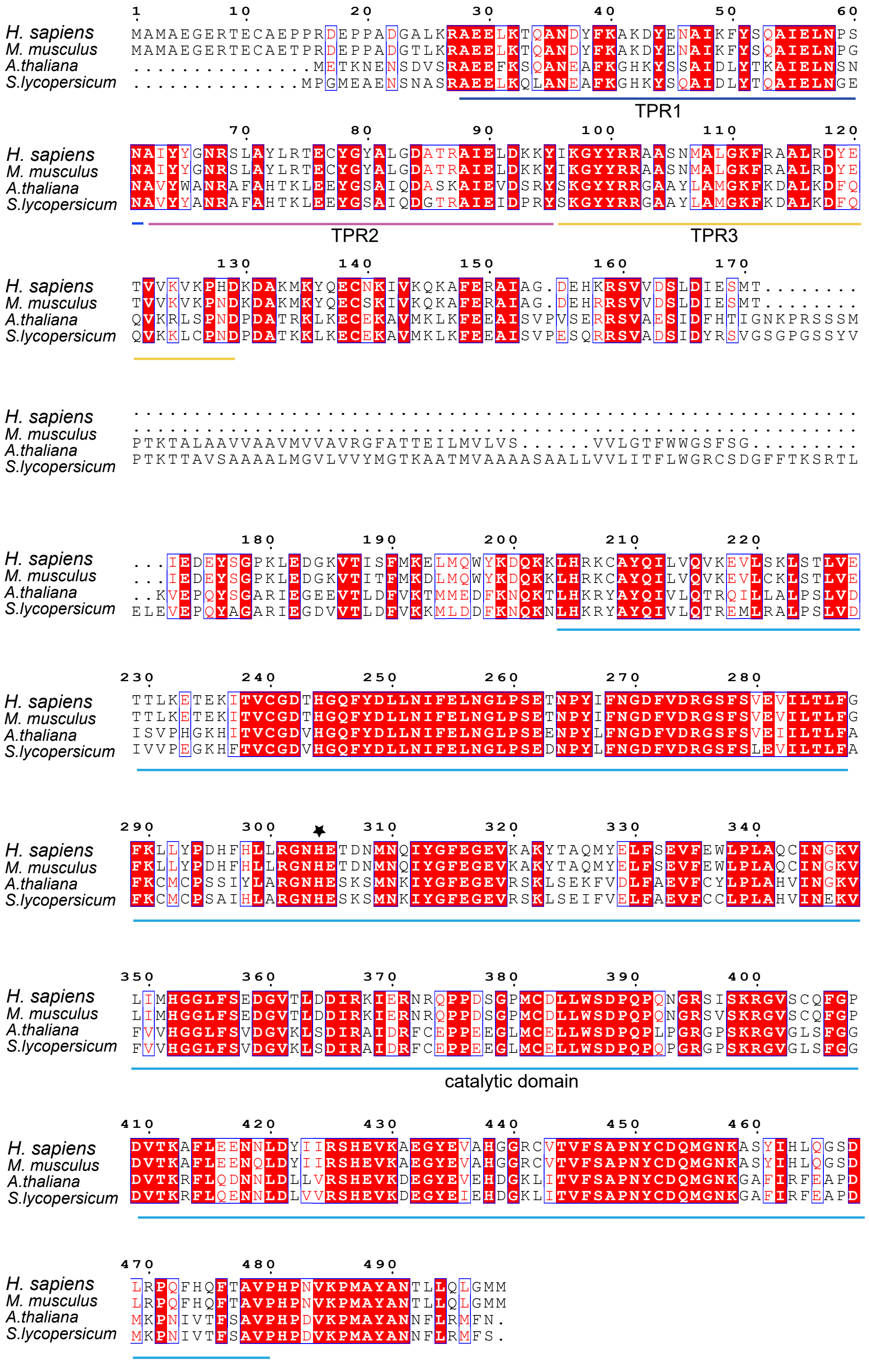

Figure S3. Multiple sequence alignment of PP5 orthologs from diverse eukaryotes.

Amino acid sequences of PP5 from human (*Homo sapiens*), mouse (*Mus musculus*), *Arabidopsis thaliana*, and tomato (*Solanum lycopersicum*) were aligned using MUSCLE. The TPR and catalytic domains are indicated by lines below the sequences and colored as follows: the TPR1 domain is in blue, the TPR2 domain is in pink, the TPR3 domain is in yellow, and the catalytic domain is in cyan.The conserved histidine residue critical for phosphatase activity (H304 in human PP5, corresponding to H344 in Arabidopsis) is highlighted with an asterisk. The alignment reveals high sequence conservation across these representative species, particularly within the catalytic domain.The figure was generated with ESPript.

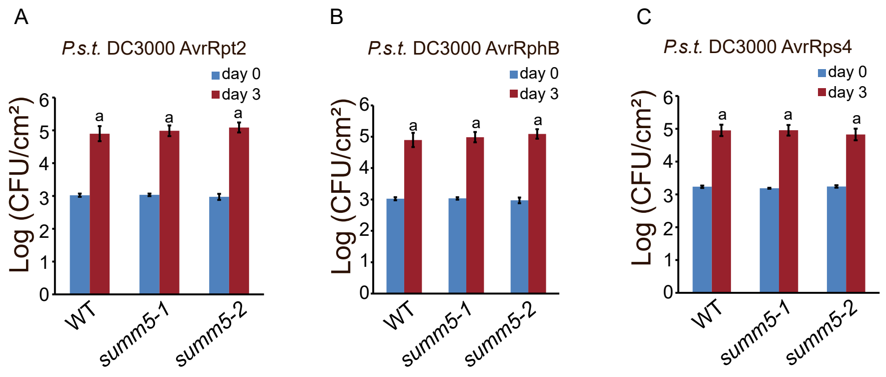

Figure S4. RPS4-, RPS2- and RPS5-mediated immunity is not affected in *summ5* mutants.
(A, B, C). Growth of bacteria in wild type (WT) and *summ5* mutant plants. Four-week-old soil-grown plants were inoculated with *P.s.t.* DC3000 AvrRpt2 (A), *P.s.t.* DC3000 AvrPphB (B) and *P.s.t.* DC3000 AvrRps4 (C). Bacterial titers at day 0 and day 3 were determined by taking leaf disks within the inoculated area. The data are shown as mean ± SD (n = 6), which was analyzed with one-way ANOVA and Tukey's test ($P < 0.01$). Labels with the same letters indicate no significant differences. The experiments were repeated twice with similar results. CFU,colony-forming units.

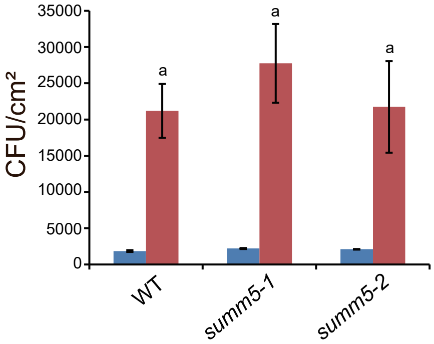

Figure S5. Growth of *P.s.t.* DC3000 *hrcC* in wild type (WT) and *summ5* mutants.
Four-week-old soil-grown plants were inoculated with *P.s.t.* DC3000 *hrcC*. Bacterial titers at day 0 and day 3 were determined by taking leaf disks within the inoculated area. The data are shown as mean ± SD (n = 6), which was analyzed with one-way ANOVA and Tukey's test ($P < 0.01$). Labels with the same letter indicate no significant differences. The experiments were repeated twice with similar results.CFU,colony-forming units.

Table S1 Primers used in this study.

**Primers**	**sequence**
SUMM2-RT-F	AGGTTCTGGACTCGGAACTAG
HA-R	gtagtctggaacatcgtatg
1305-PP5-Kpn1-F	GTGCGGGTACCTTGGTGCTACCAATGTTCAG
PP5-Xba1-R	GCTCTAGAGTTGAACATCCTGAGAAAGTTG
PP5-H344A-F	CTAGCCAGAGGAAACGCTGAAAGCAAGAGCATG
PP5-H344A-R	CATGCTCTTGCTTTCAGCGTTTCCTCTGGCTAG
PP5-Kpn1-F	GTGCGGGTACCgtctacatatctcttcgtcc
